# Glomus tumors with malignant features of the extremities: a case series

**DOI:** 10.1186/s13569-020-00142-8

**Published:** 2020-10-30

**Authors:** Taylor R. Wood, Jonathan B. McHugh, Geoffrey W. Siegel

**Affiliations:** Michigan Medicine, Rogel Cancer Center, Floor B1, Reception C, 1500 E Medical Center Dr., SPC 5912, Ann Arbor, MI 4810 USA

**Keywords:** Malignant glomus tumor, Glomus tumor with uncertain malignant potential, Glomus tumor, Tertiary sarcoma center, Case series

## Abstract

**Background:**

Malignant glomus tumors are exceedingly rare, however they can be locally aggressive and have the potential to metastasize. There is limited information available in the literature regarding treatment and outcomes, therefore we present a case series of 5 patients with glomus tumors with malignant features confined to the extremities that have presented to our tertiary sarcoma center within the last 20 years. This is the largest case series of its kind in the malignant glomus tumor literature, to our knowledge.

**Methods:**

We performed a retrospective chart review of all patients with histologically confirmed glomus tumors with malignant features in the extremities found within the University of Michigan EMERSE database since January 1st, 2000.

**Results:**

Five patients met our inclusion and exclusion criteria. Three patients were diagnosed with malignant glomus tumors, one patient with glomus tumor with uncertain malignant potential, and the last patient with malignant glomus tumor with leiomyosarcomatous features. Males and females were equally represented. Age range was 9–49 years at time of first presentation. All patients underwent an initial surgical resection. Three of the five patients (60%) underwent initial resection at an outside hospital prior to referral to tertiary sarcoma center, and all three required re-resection. One of these patients had local tumor recurrence after a planned positive margin resection and radiotherapy. Another patient had distant metastasis after a positive margin surgical resection and a short course of radiotherapy. All patients are still alive according to their medical record with the average time from initial treatment to last follow up of 59.2 months.

**Conclusions:**

Our study supports the current literature that wide-local resection with the goal of negative margins is still the current gold standard treatment for glomus tumors with malignant features. Studies with larger cohorts are necessary before recommending for or against radiotherapy or chemotherapy. Early biopsy and referral to a tertiary sarcoma center prior to surgical resection may help reduce the re-resection rate and potential seeding of the tumor in these patients, thereby improving outcomes.

## Background

Glomus tumors are rare, nearly always benign vascular neoplasms that originate from the glomus body and are usually located in the subcutaneous tissue of the extremities [1, 2]. They are typically found where glomus bodies are concentrated, such as the digits, palms, wrists, forearms, and feet, however they are most often located in the subungual layer of the fingers [3, 4]. The classic presentation is a triad of symptoms to include: hypersensitivity to cold temperature, pinpoint pain, and paroxysmal pain [2, 4]. Malignant glomus tumors are exceedingly rare, comprising as high as 2.9% of all glomus tumors [5], and are often reported in the literature as single case reports from various institutions.

Previously, glomus tumors that displayed unusual features were characterized as “atypical” or “malignant” [6]. In 2001, Folpe et. al proposed a new classification of these atypical or malignant glomus tumors, categorized as malignant glomus tumor, symplastic glomus tumor, glomus tumor of uncertain malignant potential, or glomangiomatosis [6]. This classification formed the basis for the current WHO diagnostic criterion for glomus tumors. Malignant glomus tumor is now characterized by atypical mitotic figures or marked nuclear atypia regardless of mitotic activity, and glomus tumor of uncertain malignant potential is characterized by a tumor that does not meet criteria for malignancy but has at least one atypical feature other than nuclear pleomorphism [7]. Of note, a tumor with the above-mentioned features that was over 2 cm with deep location was previously regarded as malignant, however this is no longer part of the classification [7].

Although the risk of metastasis is low, there have been reports of locally aggressive disease as well as distant metastasis [3]. Metastatic disease in particular has only been observed in malignant glomus tumors and glomus tumors of uncertain malignant potential [8], and once metastasized, the disease has a poor prognosis. One study showed 38% (8 of 21 patients) of the patients whose glomus tumors met criteria for malignancy metastasized, and of those patients with metastatic disease, 6 out of the 8 were dead from their disease after 3 years [6]. Despite this rare outcome, surgeons should be aware of the aggressive nature of these tumors prior to surgical intervention given the potential for seeding.

Wide local excision of these malignant tumors is the current gold standard of treatment, however there is a need for larger sample sizes when making conclusions about therapeutic options, adjuvant therapies, and outcomes for these rare neoplasms. Our study presents five patients with histologically confirmed glomus tumors with malignant features confined to the extremities. To our knowledge, this is the largest case series of glomus tumors with malignant features confined to the extremities in the current literature.

## Methods

Twelve patients were retrieved from the University of Michigan Electronic Medical Records Search Engine (EMERSE) database that had been coded as having a glomus tumor diagnosis from January 1st, 2000 through January 1st, 2020. A retrospective chart review was performed to identify patients whose tumors were both histologically confirmed glomus tumors with malignant features and located in the extremities. Our exclusion criteria were tumors that were not located in the extremities or that did not have histologically malignant characteristics. Clinical information collected included age at first presentation, gender, ethnicity, presenting symptoms, location of the tumor, dimensions on imaging or pathology, treatment (surgical resection, radiation, and/or chemotherapy), whether it metastasized or recurred, tumor characteristics, and distance to closest margin. These cases have not been previously reported in the literature.

## Results

### Descriptive statistics

We present five patients with histologically confirmed glomus tumors with malignant features of the extremities, along with their characteristics and outcomes. The patients’ average age was 27.4 years (range from 9–49 years) at time of first presentation. Four patients identified themselves as Caucasian (80%), and one patient identified as “Other” (20%). There was similar distribution between males (n = 2, 40%) and females (n = 3, 60%). See Table 1 for summary of patient demographics.

**Table 1:** This table represents the demographics of the five patients presented in our study

Demographics (*n* = 5)	
Average age (years)	27.4 years (4–49)
Race	80% Caucasian, 20% Other
Gender	40% Male, 60% Female

Three patients were confirmed to have malignant glomus tumors, one patient was diagnosed with glomus tumor with uncertain malignant potential, and one patient’s pathology showed a malignant glomus tumor with leiomyosarcomatous features. All five cases demonstrated areas of classic glomus morphology, at least focally, with small round cells containing central nuclei, small amounts of eosinophilic cytoplasm and clearly defined cell borders with cells growing in a perivascular arrangement in areas. All cases demonstrated cytoplasmic staining with smooth muscle actin and all cases stained with collagen IV (three of three) showed strong pericellular staining (Additional file 1: Table S1). Only Case 1′s histopathology slides were available (Additional files 2, 3), however they are representative of a classic malignant glomus tumor. Tumor locations included the calf, knee, thenar eminence, upper arm, and axilla. The tumor size was known for four of the five patients (three confirmed by MRI, one confirmed by surgical pathology). Average tumor size was 4.6 cm (range of 1.4–12 cm). Three tumors were superficial (60%,), and two were deep to fascia (40%). Four tumors demonstrated marked atypia (80%). See Table 2 for summary of tumor characteristics.

**Table 2:** This table outlines the tumor characteristics of the five patients presented in our study

Tumor characteristics (*n* = 5)	
Malignant glomus tumor	60%
Glomus tumor with uncertain malignant potential	20%
Malignant glomus tumor with leiomyosarcomatous features	20%
Average tumor size (cm)	4.625 (range 1.4–12)
Tumor depth	60% superficial, 40% deep
Marked atypia	80% yes, 20% no

All patients underwent at least one surgical resection. Three out of the five patients (60%) underwent initial resections at an outside hospital prior to referral to a tertiary sarcoma center, and two of these underwent re-resection at our institution. Two patients underwent adjuvant radiation, one of which was also recommended for adjuvant chemotherapy but followed up locally. There was one patient whose tumor metastasized (20%) and one that locally recurred (20%). All patients are still living according to their medical record. Average time from first treatment (surgical resection) to last follow up was 59.2 months (range 1–221 months). See Table 3 for a summary of oncological outcomes.

**Table 3:** This table represents the oncological outcomes of the five patients presented in our study

Oncological outcomes *(n* = *5)*	
Surgical resection	100%
Re-resection	60%
Adjuvant radiation	40%
Adjuvant chemotherapy	20%
Local recurrence	20%
Metastasis	20%
Still living	100%
Average follow up time (months)	59.2 (range 1–221)

### Case descriptions

As previously mentioned, three tumors were confirmed as malignant glomus tumors (see Additional file 1, case 1–3 for summary). Their tumor size range was 1.4–2.6 cm with an average patient age of 20–49 years. Clinical symptoms varied to include: a non-painful skin lesion on the knee; a raised, erythematous, and eventually painful skin lesion on the calf; and a prominent, non-painful mass in the thenar eminence. One patient underwent planned wide resection at our tertiary sarcoma center (Additional files 1, 2, case 1). Another patient underwent an initial resection at an outside hospital followed by wide re-resection at our tertiary sarcoma center after referral (Additional file 1, case 2). The last patient underwent an initial excision at an outside hospital followed by wide re-resection at the same outside hospital prior to referral to our tertiary sarcoma center (Additional file 1, case 3). The margins were negative in the one patient that underwent only an initial resection (Additional files 1, 2, case 1; distance to closest margin 0.4 cm).

Only one patient with confirmed malignant glomus tumor diagnosis underwent adjuvant radiation therapy (Additional file 1, case 3). Her surgery and treatment were performed at an outside hospital. Initially, she was diagnosed with sarcoma with clear cell features. Radiation therapy was recommended; however, this was stopped early once pathology was re-reviewed by another tertiary sarcoma center and confirmed as malignant glomus tumor. Six months later, she was found to have biopsy-proven metastatic disease in the lungs. She was recommended to undergo adjuvant chemotherapy, and she elected to proceed with treatment locally. None of the three patients with malignant glomus tumor diagnosis had a recurrence, and only one developed metastatic disease.

One patient’s pathology revealed glomus tumor of uncertain malignant potential (Additional file 1, case 4). Her tumor size was 12 cm and she was 32 years old at first presentation. Clinical symptoms began with paresthesias in her fingers and hand with eventual paresis of the thumb. Of note, her tumor involved the median nerve in the upper arm and went undiagnosed for nearly 5 years despite these symptoms. Eventually, a mass was palpated which prompted an MRI. She underwent surgical resection for initial treatment. Margins were planned positive in order to spare median nerve function. She then underwent post-operative radiation therapy given planned positive margins. The tumor was deep. No chemotherapy was recommended. No metastasis was noted, but the tumor did recur locally 18 years later. She underwent wide surgical resection without any further adjuvant therapy.

The final patient’s pathology confirmed malignant glomus tumor with leiomyosarcomatous features (Additional file 1, case 5). No tumor size was available, and she was 9 years old at time of first presentation. Clinical symptoms included an enlarging mass located in the axilla without pain or tenderness. The patient underwent initial resection at an outside hospital prior to referral to a tertiary sarcoma center. She then underwent wide re-resection at our institution once pathology was confirmed. The tumor was deep with “unclear” margins. She did not undergo adjuvant radiation or chemotherapy. There has been no recurrence or metastasis to date.

## Discussion

Malignant glomus tumors are exceedingly rare with limited information available in the literature regarding treatment and outcomes. Although a rare occurrence, malignant glomus tumors and glomus tumors of uncertain malignant potential have been shown to both be locally aggressive and have the potential to metastasize [3, 9, 10]. Once metastasis occurs, prognosis is poor [6, 8]. Wide local excision is the current gold standard treatment option given low potential for glomus tumors with malignant features to metastasize, however surgeons should be privy to this potential outcome prior to surgical intervention [11]. Our study presents five patients with glomus tumors with malignant features of the extremities along with their treatment and outcomes. This is the largest case series of its kind in the current literature, to our knowledge.

Three patients were confirmed to have malignant glomus tumors, one was diagnosed with glomus tumor with uncertain malignant potential, and the last patient’s diagnosis was malignant glomus tumor with leiomyosarcomatous features. There was an even distribution of tumors in the upper and lower extremities. 60% of the tumors were superficial to fascia. Our study supports the current literature by showing no predominant gender associated with glomus tumors with malignant features as well as a wide range of age groups affected.

All patients underwent a surgical resection, however four of these were initially performed at outside hospitals prior to referral to our tertiary sarcoma center. Of these four patients, three needed to undergo re-resection. Two of these re-resections were performed at a tertiary sarcoma center after glomus tumor with malignant features was diagnosed. With a 60% re-resection rate after histopathology confirmed glomus tumor with malignant features, we suggest early referral to a tertiary sarcoma center as well as obtaining a biopsy of these suspicious lesions, if feasible, prior to proceeding with an initial surgical resection. These interventions could potentially decrease the re-resection rate, leading to a reduction in surgical costs, morbidity, and a lower potential for seeding the tumor by having the correct diagnosis for proper surgical planning.

There was a 20% recurrence rate in our series of five patients as well as a 20% metastasis rate. The only tumor recurrence was in a patient with planned positive margin surgical resection despite undergoing adjuvant radiation therapy. The only patient with metastatic disease underwent two resections at an outside hospital that resulted in positive margins prior to referral and histopathological confirmation by a tertiary sarcoma center. 60% of our patients did not have recurrence or metastatic disease after surgical resection or re-resection confirmed negative margins. This supports the current literature that wide resection of these glomus tumors with malignant features to achieve negative margins should be the primary goal and treatment option, if feasible.

Two of our patients did receive adjuvant radiation therapy. One of these patients underwent radiation treatment prior to definitive diagnosis of a glomus tumor with malignant features, therefore radiation was stopped early once the diagnosis was made. The other patient received adjuvant radiotherapy due to a planned positive margin surgical resection to preserve median nerve function. This patient had tumor recurrence nearly 18 years later despite adjuvant radiation therapy. Based on the limited amount of data available in the malignant glomus tumor literature, our results support the observation that radiotherapy may offer little long-term benefit to these patients [12].

Chemotherapy was only offered to one patient that developed metastatic disease, however she elected for treatment at a local facility. There is little information regarding effective chemotherapy regiments for these tumors, therefore providers tend to use treatments similar to what has been effective for sarcomas [12]. Given that none of our patients received chemotherapy, we cannot come to any further conclusions regarding the option of chemotherapy for patients with glomus tumors with malignant features.

Several limitations exist in our study. This is a retrospective case series, therefore we are simply reporting outcomes and not testing these conclusions prospectively against a control group. Additionally, our sample size of five patients is small, as these were the only five patients diagnosed with glomus tumors with malignant features that have been seen at our tertiary sarcoma center in the last 20 years. We are also limited by what was documented in the medical record, therefore these patients could have had a treatment or outcome that we may not know about if they presented to an outside hospital system.

## Conclusions

We presented five patients diagnosed with glomus tumors with malignant features in the extremities who presented to a tertiary sarcoma center within the last 20 years. Our findings support the current literature of wide resection with a goal of negative margins as the gold standard treatment options for these patients. We suggest that early biopsy and referral to tertiary sarcoma center prior to an initial surgical resection would decrease the re-resection rate as well as the potential for recurrence or metastatic disease. Little data exists regarding effectiveness of radiotherapy or chemotherapy for these patients, therefore future studies with larger cohorts are needed to explore these treatment options as potential benefit for these patients.

## Supplementary information

**Additional file 1.** Summary of Patient Data. This table presents the demographics, tumor characteristics, and oncological outcomes individually for each of the five patients.

**Additional file 2.** Representative Histology. This represents a low-power histopathology slide demonstrating malignant glomus morphology.

**Additional file 3.** Representative Histology. This represents a high-power histopathology slide demonstrating small round cells containing central nuclei, small amounts of eosinophilic cytoplasm and clearly defined cell borders with cells growing in a perivascular arrangement in areas.

## Data Availability

The data that support the findings of this study are available from the University of Michigan Electronic Medical Records Search Engine (EMERSE) but restrictions apply to the availability of these data, which were used under license for the current study, and so are not publicly available. Data are however available from the authors upon reasonable request and with permission of Michigan Institute for Clinical and Health Research (MICHR) at the University of Michigan.
