# Supplementary figures and images for: Glomus tumors with malignant features of the extremities: a case series
Source: Clin Sarcoma Res. 2020 Oct 30;10:20. doi: 10.1186/s13569-020-00142-8 (PMC7596933; doi:10.1186/s13569-020-00142-8)

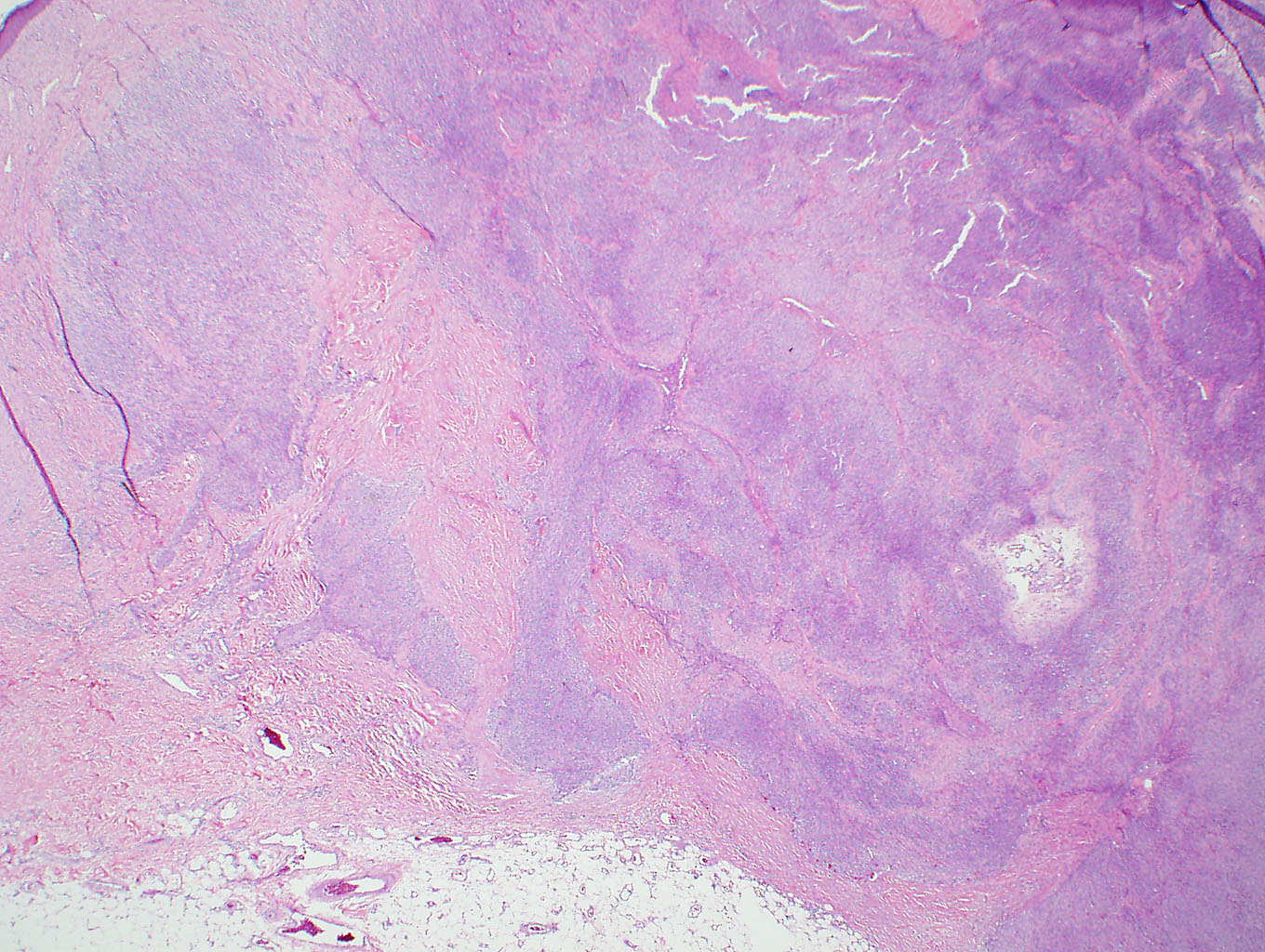

Supplement: Supplementary file 2 — Additional file 2. Representative Histology. This represents a low-power histopathology slide demonstrating malignant glomus morphology. [file 13569_2020_142_MOESM2_ESM.jpg]

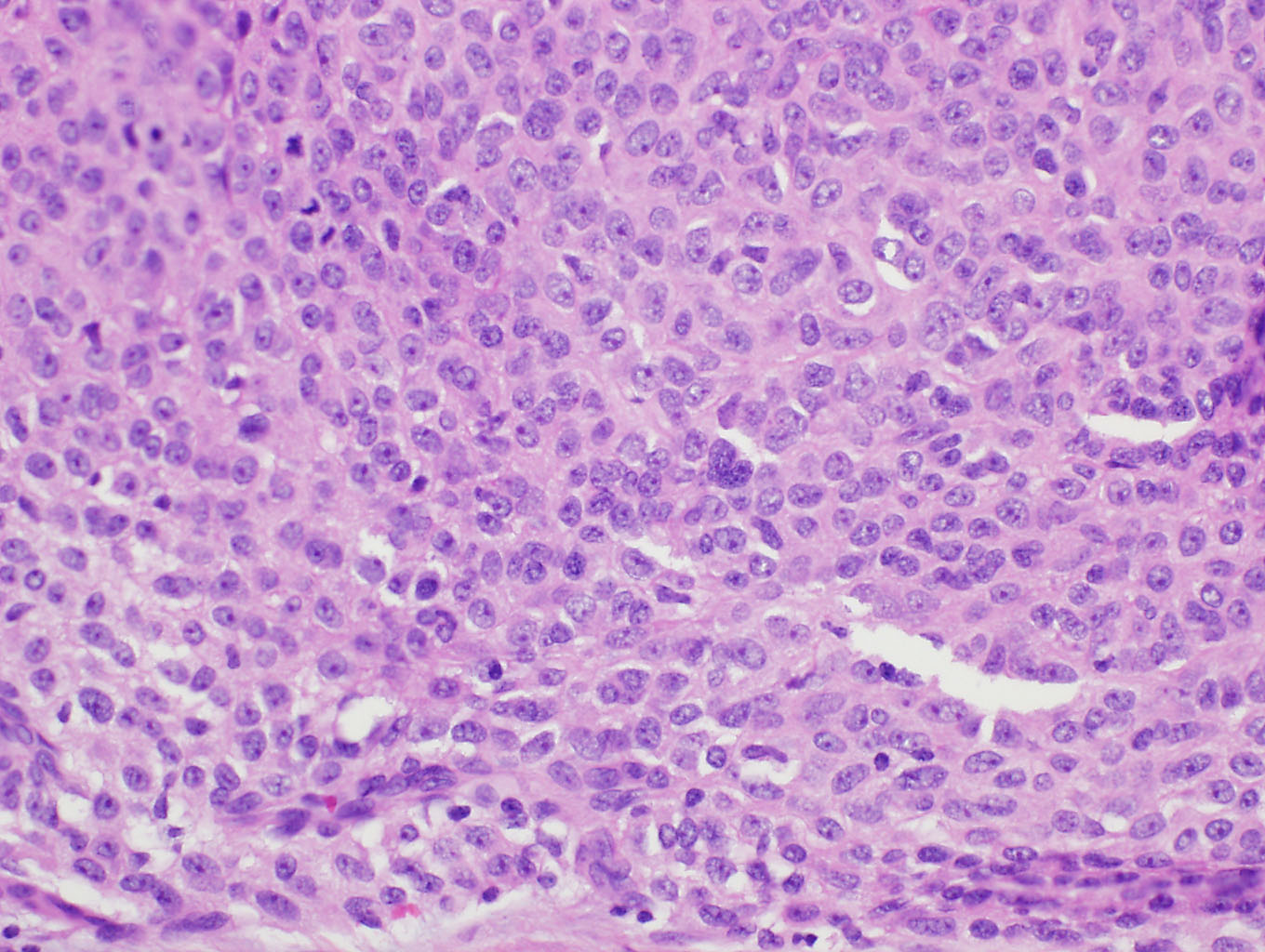

Supplement: Supplementary file 3 — Additional file 3. Representative Histology. This represents a high-power histopathology slide demonstrating small round cells containing central nuclei, small amounts of eosinophilic cytoplasm and clearly defined cell borders with cells growing in a perivascular arrangement in areas. [file 13569_2020_142_MOESM3_ESM.jpg]
